# Through the Eyes of Creators: Observing Artificial Molecular Motors

**DOI:** 10.1021/acsnanoscienceau.1c00041

**Published:** 2022-01-13

**Authors:** Ivan N. Unksov, Chapin S. Korosec, Pradheebha Surendiran, Damiano Verardo, Roman Lyttleton, Nancy R. Forde, Heiner Linke

**Affiliations:** † Solid State Physics and NanoLund, Lund University, Box 118, SE-221 00 Lund, Sweden; ‡ Department of Physics, Simon Fraser University, V5A 1S6 Burnaby, British Columbia, Canada; § AlignedBio AB, Medicon Village, Scheeletorget 1, 223 63 Lund, Sweden

**Keywords:** artificial molecular motors, optical microscopy, FRET, optical tweezers, magnetic tweezers, atomic force microscopy, scanning tunnelling microscopy

## Abstract

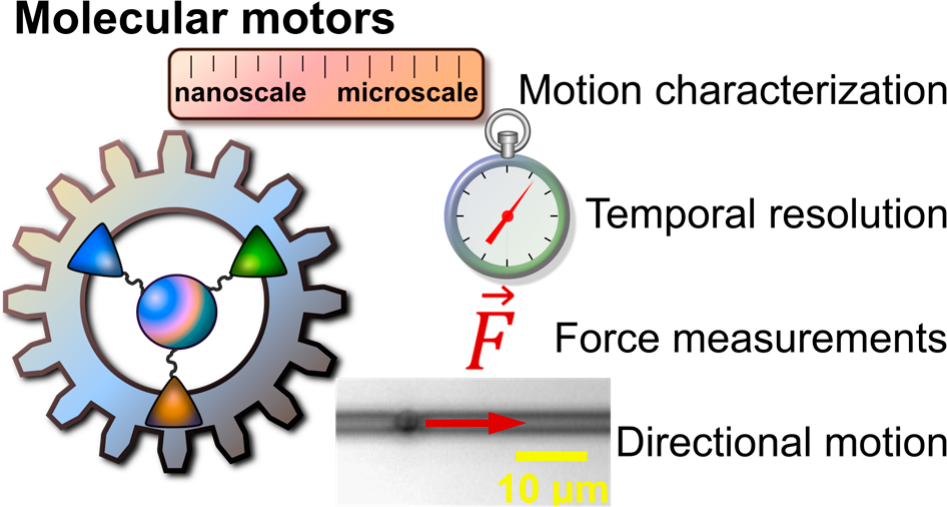

Inspired by molecular motors in biology, there has been significant progress in building artificial molecular motors, using a number of quite distinct approaches. As the constructs become more sophisticated, there is also an increasing need to directly observe the motion of artificial motors at the nanoscale and to characterize their performance. Here, we review the most used methods that tackle those tasks. We aim to help experimentalists with an overview of the available tools used for different types of synthetic motors and to choose the method most suited for the size of a motor and the desired measurements, such as the generated force or distances in the moving system. Furthermore, for many envisioned applications of synthetic motors, it will be a requirement to guide and control directed motions. We therefore also provide a perspective on how motors can be observed on structures that allow for directional guidance, such as nanowires and microchannels. Thus, this Review facilitates the future research on synthetic molecular motors, where observations at a single-motor level and a detailed characterization of motion will promote applications.

## 1 Introduction

Molecular motors, also commonly referred to as molecular machines, are molecules or supramolecular entities capable of moving without an external force applied. They achieve this by harnessing a source of energy, for example, in a chemical, thermal, photonic, or particle (e.g., electrons) form. Artificial motors are often synthesized to mimic their natural analogues such as myosins and kinesins (walker motors) or rotary ATPases (ATP = adenosine triphosphate), and the study of them helps to gain a better understanding of those natural motors and, more generally, of energy conversion at the nanoscale. Moreover, some future artificial motors may ultimately offer advantages over biological molecular motors for certain applications, for example, a higher speed or the ability to operate in a wider range of environments. The field has recently seen a lot of growth, with multiple research articles published just in the last three years describing novel synthetic motors.^1−8^

The motors implemented so far have often been designed for a specific function, such as molecular switches and rotors,^7,9−12^ transport of cargoes,^13,14^ recording digital information,^15^ employing quantum tunnelling for directional motion,^16^ phototherapy of cancer,^17^ controlled supramolecular aggregation,^18^ governing cellular processes,^19^ and energy-efficient performance at low temperatures.^20^ In addition, artificial motors could be an advantageous alternative to their natural counterparts that are currently employed in emerging applications such as highly energy-efficient biocomputing devices.^21,22^ It is also possible to modify natural motors by adding functional groups to the molecules or by using synthetic scaffolds for the motion of the natural motors.^23,24^

Existing reviews on this subject generally focus on the design of artificial motors^25^ or on a specific variety of motors, chosen by the type of motion: molecular walkers,^26,27^ rotary motors;^28,29^ by building material, for example, DNA^30−33^ or proteins.^34^ However, the wide range of methods available for the characterization of molecular motors has not, to our knowledge, been the subject of a comprehensive review.

Since the earliest studies, spectroscopy has been used to provide evidence of the action of synthetic motors at the ensemble level in bulk solution. Motors are still commonly studied using fluorescence spectroscopy,^8,35,36^ nuclear magnetic resonance (NMR),^6,15,20,37−40^ circular dichroism,^10,15^ UV–visible spectroscopy,^40^ and matrix-assisted laser desorption/ionization time-of-flight (MALDI–TOF) mass spectrometry.^14^ These spectroscopy techniques have historically been the most accessible in terms of setup availability and sample preparation.

However, ensemble techniques generally provide no or limited information about motor processivity, directionality, and speed or the generated force. To determine these quantities, single-molecule techniques are generally required, and spectacular progress in this area has been made in recent years, such as the development of super-resolution microscopy^41−47^ and high-speed atomic force microscopy (AFM).^48−51^

Here, we review the most important techniques allowing for a single-molecule characterization of artificial molecular motors. Our aims are to provide the experimentalist (i) with an overview of available techniques, (ii) with a guide to the literature where examples of their use can be found, and (iii) with a tool to choose the most suitable technique for a given type of motor and characterization purpose.

**Figure 1:**
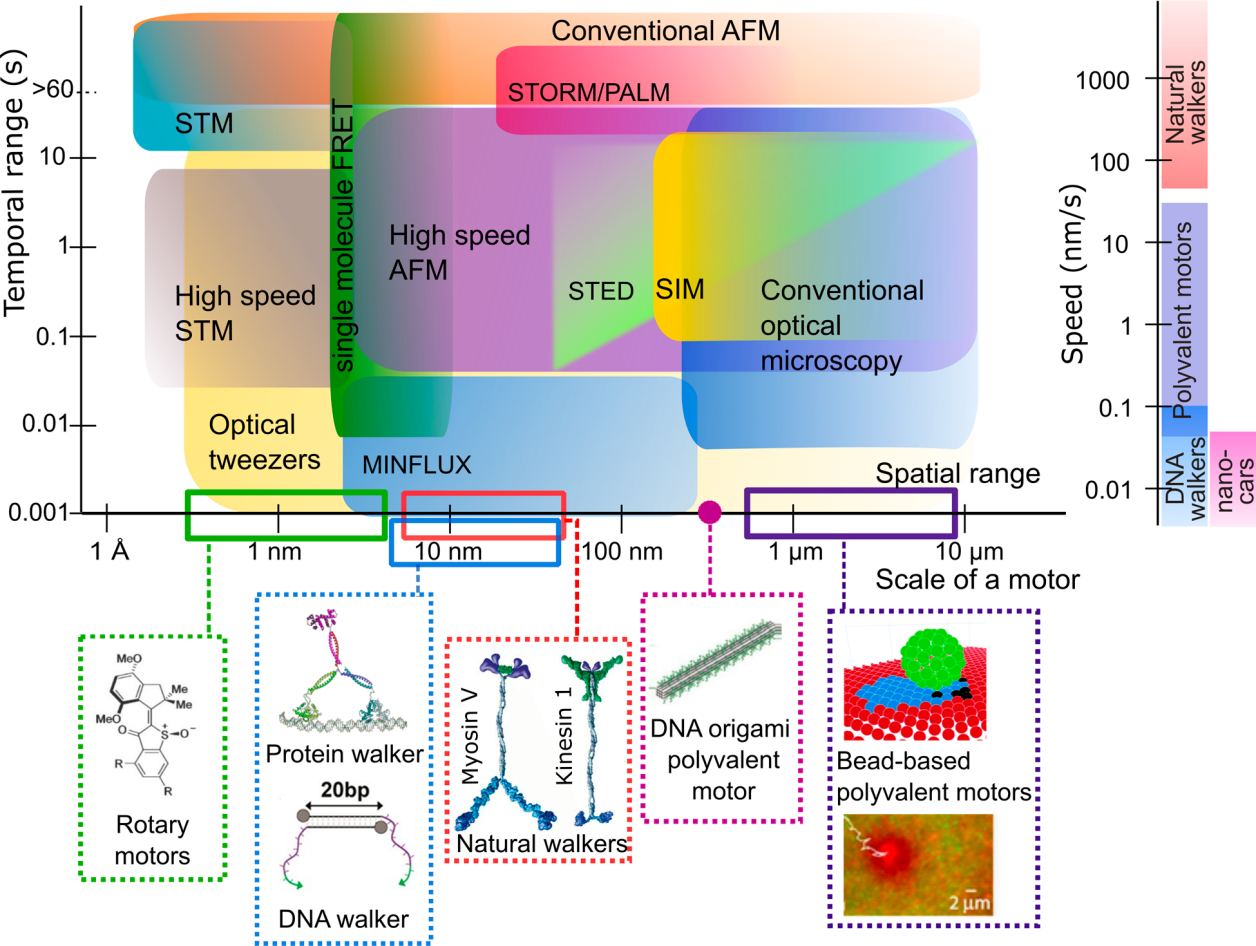
Typical spatiotemporal range achieved using experimental techniques, scale of exemplary molecular motors (or their moving components), and speed of motor motion. Speeds are based on refs (60−63) for natural molecular motors, on refs (1, 4, and 64−67) for polyvalent motors, on ref (68−71) for DNA walkers, and on refs (72 and 73) for “nanocars”. Drawing of the rotary motor is reproduced from ref (11). Copyright 2020 American Chemical Society. Protein walker adapted from ref (74). Copyright 2017 American Chemical Society. DNA walker adapted from ref (68). Copyright 2016 American Chemical Society. DNA origami motor adapted from ref (1) with permission from John Wiley and Sons. Copyright 2020. One bead motor (top) reproduced from ref (3) with permission. Copyright 2020 Royal Society of Chemistry. Another bead motor (bottom) reprinted from ref (75) with permission from PNAS. Natural motors adapted with permission from ref (76). Copyright 2003 Elsevier.

For an overview of the field of artificial motors and the demands on performance characterization, it is useful to consider the spatial and temporal scale of the motor motion (Figure 1), each spanning several orders of magnitude. The smallest motors are rotary constructs that typically rely on an isomeric rotation around a molecular bond and are typically ∼1 nm in size.^19,28,52−56^ Another class of artificial motors is designed for motion across a surface; these motors are referred to as walkers and are generally larger than rotary motors (several to tens of nanometers). At the largest end of the scale one finds polyvalent (i.e., with multiple functional molecular units) motors based on micron-scale beads.

Here we focus on techniques to analyze the motion of artificial molecular motors that walk or diffuse across their landscape, although we overview a number of molecular rotors in connection to relevant methods. For a more comprehensive outlook on small rotary motors, we address the interested reader to the reviews.^28,29,57^ The scale of the motion events (e.g., steps) that need to be observed correlates with motor size and thus also varies from the nano- to the microscale distances, depending on the motor type (Figure 1). Likewise, the time scale of the motion to be observed varies widely, from the microsecond^58,59^ and millisecond^60^ scale of stepping of natural molecular motors to minutes or hours of motion for some polyvalent motors and DNA walkers. Currently, artificial molecular motors of the walker type are typically slower than their natural counterparts.

For this wide variation of requirements, Figure 1 also provides an overview of the spatiotemporal range of commonly used characterization methods. The most common methods employed for many motor types are microscopy techniques and Förster resonance energy transfer (FRET). In Sections 2.1 and 2.3, respectively, we review optical and atomic force microscopy. FRET, reviewed in Section 2.2, does not visualize the motion but allows one to observe changes in inter- or intramolecular distances with outstandingly high spatial resolution, and it is therefore widely used to probe and characterize motor dynamics. Section 2.4 is dedicated to scanning tunnelling microscopy (STM), with examples of its usage for characterization of small molecular motors. Optical/magnetic tweezers, reviewed in Section 2.5, allow one to measure forces generated by motors. We do not review scanning/transmission electron microscopy (SEM/TEM) and related techniques because those have limited applications to the characterization of artificial molecular motors.

A part of our Review describes a methodology of experiments with Brownian burnt-bridges motors. This vast class of motors is introduced in Section 3.1. In Section 3.2, we address data analysis approaches to these and other motors, for which individual steps cannot be resolved, and where motor trajectories may not be one-dimensional (1D). We describe how the characterization of motors can be done using a mean squared displacement (MSD) analysis, that is, how an ensemble-averaged or time-averaged displacement can be employed to estimate the anomalous diffusion exponent, speed, and processivity of the motor.

Looking ahead to making artificial molecular motors useful, it will often be necessary to guide the motion of these motors along a nanofabricated track as well as to characterize such guided motion. In Sections 4.1 and 4.2, we present some results on the use of microfabricated channels and nanowires for this purpose and showcase their advantages and underlying challenges.

Finally, we provide an outlook on the future use of force and distance measurements for characterizing artificial molecular motors, for example, by optical or magnetic tweezers, which are commonly used for studies of natural molecular motors.

## 2 Characterization Methods

### 2.1 Optical Microscopy

Optical microscopyis the most widely used method for the tracking of molecular motors. Its versatile modalities are employed for imaging in bulk liquid as well as on surfaces and cover a broad range of spatiotemporal scales (see Figure 1 and Table 1 for overviews). However, for nanoscale motors it is important to keep in mind that conventional optical microscopy is diffraction-limited. Here we will first describe how conventional microscopy can be applied to observe artificial motors, and then we will outline super-resolution approaches that allow one to resolve events beyond the diffraction limit.

**Table 1:** Methods Typically Used in Molecular Motor Studies

method	spatial resolution (lateral)	temporal resolution/acquisition time	measurements	label-free options
Conventional optical microscopy	Limited by diffraction	Limited by detector, ∼ms for a typical EMCCD/CMOS camera,^207^ except for high speed instruments.^208^ For fluorescence, theoretically limited by excitation–emission cycle (∼ns^209^)	Position; compatible measurement modalities: distance 2–10 nm – **single-molecule FRET**; fluorescence lifetime – **FLIM**;^210,211^ diffusion rate – **FRAP**,^210^**FCS**^212^	Brightfield/darkfield, contrast, polarization, Raman scattering^213^
Super-resolution optical microscopy	**SIM**: increased twofold compared to diffraction-limited value;^45^	**SIM**: up to video rate;^110,214−216^	Position, super-resolution methods are compatible with **FRET**,^218^**FLIM**,^219^**FCS**^220^	Most super-resolution methods rely on fluorescence. However, label-free options are emerging, e.g., based on Raman scattering microscopy combined with **STED**, **SIM**, etc., reviewed in refs (221 and 222)
	**STED**: 30–60 nm;^113,115^	**STED**: <50 ms for a μm-sized field of view,^114^ seconds for a 10 μm-sized field of view;^217^		
	**STORM** and similar: ∼20 nm spatial resolution;^47,111^	**STORM**: minutes		
	**MINFLUX**: <5 nm (up to size of fluorophore);^44,105^	**MINFLUX**: <0.5 ms for <20 nm resolution;^44^		
	**DNA-PAINT**: <10 nm^46^	**DNA-PAINT**: minutes to hours^46^		
AFM	**Conventional AFM**: sub-nm; **HS-AFM**: ∼nm^51^	**Conventional AFM**: >1 min; **HS-AFM**: 20–100 ms^50,51^	Position; force spectroscopy down to pNs	**AFM** is typically label free
STM	sub-nm	**Conventional STM:** ∼min;^54^**fast STM:** <1 s^150−152^	Position; tunnelling current spectroscopy	**STM** is label-free but requires a conductive sample/surface
Optical/magnetic tweezers	**OT**: sub-nm;	Typically 0.01–1 s,^156,157,223−225^ up to μs-scale in some setups^58^	Sub-pN forces; distance	**OT/MT** require particles for trapping, and the trapped objects must be attached to those particles
	**MT**: 2–10 nm^157,223^			

As formulated by Abbe, an optical setup can resolve two objects if their lateral separation is larger than an Airy disk radius , where λ is the wavelength of the light used for observation, and NA is the numerical aperture of the objective. For light in the visible range, *r*_A_ is a few hundred nanometers. Thus, a single object smaller than *r*_A_ appears in the microscope as a single diffraction-limited spot, the central region of which will have radius *r*_*A*_. A single spot is also observed when multiple objects are located within *r*_A_.

Conventional optical microscopy is well-suited for motors based on beads or similar opaque or refractive particles, for which label-free detection is possible, most simply using brightfield imaging. Brightfield microscopy was used to observe the enzymatically driven motion of polystyrene microspheres.^65,77^ Another accessible option for label-free detection is darkfield microscopy, where only the light scattered by a sample enters the objective, which may offer a higher contrast. A range of darkfield microscopy approaches, including tracking and distance measurements, have been recently reported^78^ for plasmonic particles. There is also a plethora of other label-free modalities,^79^ for example, interferometric scattering microscopy^80^ and nonlinear imaging.^81,82^

**Figure 2:**
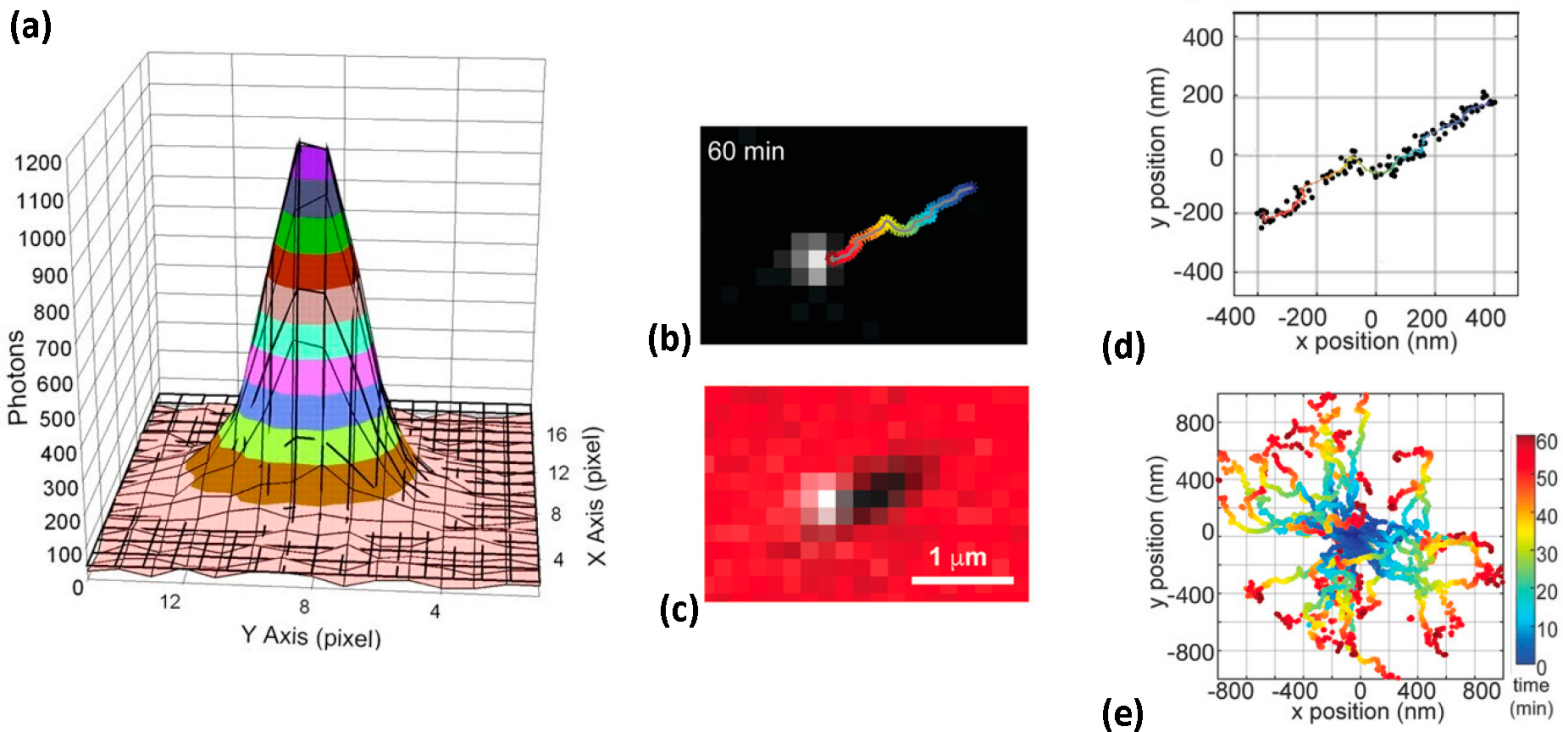
(a) Single-fluorophore localization with the FIONA approach: in this example, the center of the Gaussian fit (solid lines) of a point-spread function (PSF) (colorful), imaged with TIRF, was localized with 1.3 nm precision using a total number of 14 200 detected photons (0.5 s integration time). From ref (86). Reprinted with permission from AAAS. Copyright 2003. (b–e) Localization of individual molecular motors: trajectory of a DNA origami molecular motor tracked using a fluorescence signal from the motor (b) and overlay (c) of signals from the motor and track; subdiffraction localization of a single (d) and multiple (e) motors. Reproduced with permission from ref (1). Copyright 2020 John Wiley and Sons.

Motors of molecular sizes are usually labeled to be observed using fluorescence microscopy. For motors of subdiffraction size, fluorescence microscopy with a subsequent localization of the motors in the images is an option. Localization implies that the point-spread function in the diffraction-limited image of the object is fitted, and the center of the distribution indicates the actual position of the object (Figure 2a). As outlined in ref (83), the localization accuracy is approximately proportional to 1/√*N*, where *N* is the number of photons collected from the fluorophore. The key challenge then is to collect as many photons per position as possible before photobleaching (the light-induced loss of fluorescence properties of the fluorophore) occurs.^84,85^ In an early work, Yildiz and co-workers^86^ used this approach, nicknamed FIONA (fluorescence imaging with one-nanometer accuracy), to trace the steps of a myosin motor along actin with 1.5 nm precision and with a time resolution of 0.5 s, as shown in Figure 2a. For artificial motors, the tracking of molecular spiders on DNA origami was described with experimental details in ref (87). More recently, 50 nm nanoparticles functionalized with DNA by Salaita and co-workers were tracked using a localization approach, obtaining the speed (up to 50 nm/s) and processivity of the motors.^4^ Fluorescence microscopy with localization (Figure 2b–e) was also performed by the same group on 130 nm DNA origami motors.^1^

A limiting factor in conventional epifluorescence microscopy is the background signal from other fluorescent molecules in solution. In total internal reflection fluorescence (TIRF) microscopy, the fluorescence is excited by evanescent waves selectively within a layer of only ∼100 nm thickness above a glass coverslip, leaving all other fluorescent molecules dark. This can drastically improve the signal-to-noise ratio and enable a high vertical spatial resolution at an unchanged lateral resolution;^88^ however, an exponential decrease in the fluorescence excitation with vertical distance^89^ needs to be taken into account in intensity measurements. This *z*-dependence of TIRF has been utilized for three-dimensional (3D) microscopy reaching nanometer resolution along the *z*-axis.^90^ An additional capability of TIRF microscopy is that the incident light can be polarized parallel or perpendicular to the glass coverslip. Because the probability of photon absorption is a scalar product of the orientation of a fluorophore relative to the polarization of evanescent field, one can use polarization to achieve a selective excitation of fluorophores of a specific orientation.^91−93^ This technique is promising for dynamical studies of artificial molecular motors, similar to how it was used to observe short-lived states in the motion of myosin V on a 10–15 ms time scale.^59^ The features of two major types of setups, namely, the prism-based TIRF introduced in (94) and the more frequently used objective-based TIRF described in (95), are reviewed in refs (88, 96, and 97). Because TIRF is effective in reducing an out-of-focus signal, it is often a method of choice for single-molecule FRET and the subdiffraction localization of labeled molecular motors. TIRF has been employed for the imaging of a DNA walker^70^ on a DNA track, for single-molecule FRET studies of a biohybrid DNA rotor–stator nanoengine,^98^ and a reconstructed kinesin-2-powered intraflagellar-transport complex.^99^ TIRF has also been widely used for a nanometer-precision localization and tracking of natural molecular motors: myosins and kinesin using the FIONA approach^86,100^ and, more recently, myosins.^101,102^ Despite the limited penetration depth, TIRF has also been used to visualize microscale motors operating near a coverslip surface.^103^

A range of specialized super-resolution techniques allows one to achieve a subdiffraction resolution (see also Table 1). These include stimulated emission depletion (STED) microscopy,^42^ stochastic optical reconstruction microscopy (STORM),^47^ photoactivated localization microscopy (PALM),^41^ fluorescence photoactivation localization microscopy (FPALM),^104^ structured illumination microscopy (SIM),^45^ MINFLUX (which minimizes fluorescence fluxes),^43,44,105^ or DNA points accumulation for imaging in nanoscale topography (DNA-PAINT).^46,106^ These methods have been used for natural motors: PALM for imaging of the SpoIIIE DNA pump,^107^ STORM for dyneins,^108^ and TIRF-SIM for filament transport studies.^109,110^ However, as of yet, super-resolution methods have not been widely applied to artificial motors, although their capabilities would be useful for tracking nanosized motors. One example of a case where super-resolution methods should be preferred to a localization from a conventional imaging is the high density of fluorophores: in such a sample, localization is only possible if subsets of the fluorophores are switchable, which is realized in STORM/PALM. Another example is a nanosized motor system where only a limited number of photons can be detected before fluorophores on the motor move due to the motor motion or undergo photobleaching; MINFLUX, in which localization relies not on a camera but on the targeting of the excitation beam, can in this case minimize the needed number of photons and maximize the temporal resolution up to a sub-millisecond scale. One more example is the recent SIM measurement of the width, estimated as 133 ± 43 nm, of tracks of fluorescence depletion made by a DNA origami nanomotor.^1^

However, it is important to keep in mind that the resolution of these methods is also limited. STORM reaches ∼20 nm spatial resolution^47,111^ but at the cost of long imaging times (order of minutes). A STED setup allows for 30–60 nm resolution for suitable fluorophores, among which red fluorophores, stimulated with near-IR wavelengths, may be preferred.^112,113^ That level of resolution has been achieved at a video rate for a micron-sized field of view,^114^ but imaging slows down for larger fields of view where the scanning speed is a limitation. SIM improves the diffraction-limited resolution twofold^45,115^ and may reach a subsecond temporal resolution.

The highest spatial resolution obtained (less than 5 nm, which is on the resolution limit imposed by the size of a fluorophore label) was achieved by the MINFLUX^43^ and DNA-PAINT.^46,106^ For DNA-PAINT, which relies on short DNA strands for labeling and imaging, a resolution this high requires up to hours of acquisition time,^46^ which limits its potential for observing molecular motors. In contrast, MINFLUX has recently achieved an outstanding temporal resolution of less than 0.5 ms^44,105^ for a nanometer-scale spatial resolution, which makes it promising for nanoscale molecular motors, although in the case of fast motion tracking the MINFLUX localization was reported to be somewhat less precise, for example, tens of nanometers.^43,44^

An outstanding sub-millisecond temporal resolution was achieved in tracking natural kinesin motors as they passed through a small volume of excitation and detection in a confocal setup.^116^ However, regular confocal setups are uncommon for microscopy studies on artificial molecular motors, so we do not describe the methodology here.

For future subdiffraction studies on small molecular motors, the reconstruction of microscopy images using deep learning algorithms is promising. A content-aware restoration of images was reported for denoising and reconstruction of object positions with subdiffraction accuracy.^117,118^ Another deep learning approach was presented for denoising and enhancing the resolution of TIRF and confocal images to the resolution of SIM and STED, respectively.^119^

To summarize, optical microscopy features a combination of high temporal resolution and spatial resolution of a few hundred nanometers (at a conventional setup). Localization or super-resolution methods are necessary to achieve nanometer accuracy, but many of those come at the cost of a lower temporal resolution. In the following we discuss FRET, AFM, and STM, which are not limited by diffraction.

### 2.2 Förster Resonance Energy Transfer (FRET)

FRET is the nonradiative energy transfer between fluorophores, from an excited donor to an acceptor. In FRET experiments one can determine nanometer-scale distances between the donor and acceptor by measuring the fluorescence from both. The efficiency of FRET depends on the distance, being inversely proportional to , where *r* is the distance between donor and acceptor, and *R* represents the 50% efficiency distance.^120^ A larger overlap between the acceptor excitation and donor emission increases the FRET efficiency; however, to avoid crosstalk between the two dyes, the overlap of donor and acceptor excitation bands should be minimal as well as that of donor and acceptor emission.^121^ FRET enables measurements of typically 2.5–10 nm distances between fluorophores involved in a FRET pair.^122,123^ FRET in a bulk solution requires a synchronization of multiple molecular events, which may issue its application for molecular motors. A single-molecule FRET typically employs a microscope, and its temporal resolution is limited by a camera or a photon counter. For example, a single-molecule FRET with a 100 μs temporal resolution has been achieved using a fast counter in ref (124).

FRET can be performed with multiple excitation lasers: in alternating laser excitation (ALEX) FRET,^125^ one laser excites the donor, and the other directly excites the acceptor. It is then possible to rule out crosstalk due to a spectral overlap between the donor and acceptor. ALEX FRET can be used to observe multiple fluorophore species.^33^ For molecular motors, a related pulsed interleaved excitation (PIE) technique has been used to observe a DNA tweezer molecular machine:^126^ excitation with two alternating lasers allowed for FRET measurements while guaranteeing that the observed motor had both a donor and acceptor on it.

FRET experiments on artificial molecular motors typically use fluorophores that are positioned either on different parts of a motor or on the motor and substrate. This approach allows one to detect a motor operation in a bulk solution. For example, for DNA walkers^127,128^ that move due to the hydrolysis of single-stranded DNA serving as a fuel, the kinetics were investigated by the attachment of a FRET pair on the motor and fuel and observing the FRET in the bulk.

**Figure 3:**
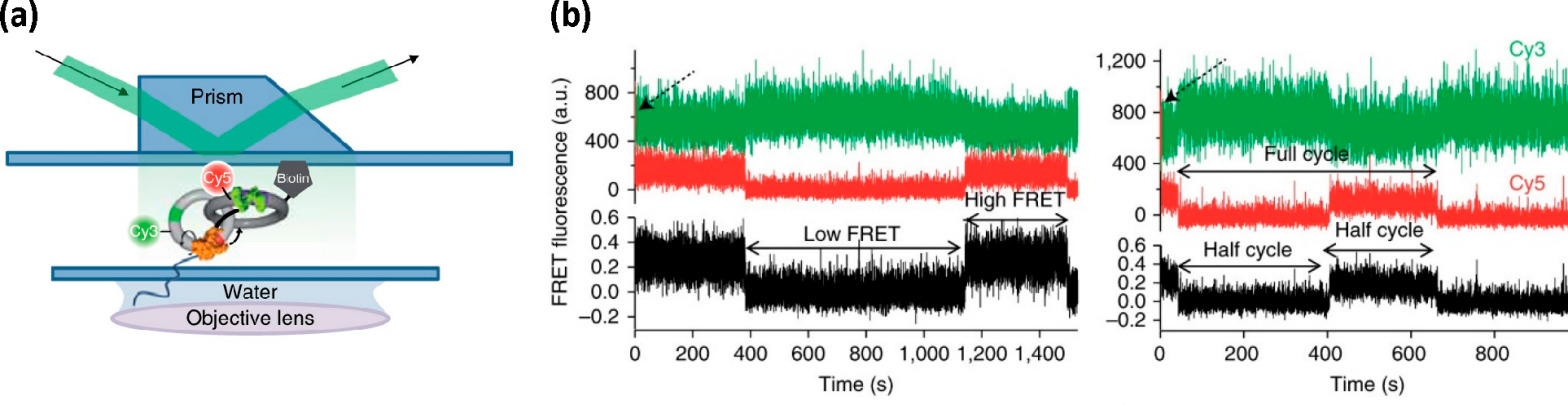
Single-molecule FRET observed for a DNA catenane motor (a) using TIRF. FRET intensity (b) of Cy3 donor (green line), Cy5 acceptor (red line), and the resulting FRET signal (black line). Cycles of rotation of the motor are indicated by periodic changes of the signal. Direct excitation of the acceptor confirmed its presence at the start of the measurement (black arrow). Reprinted by permission from Springer Nature from ref (98). Copyright 2018.

Single-molecule (single-particle) FRET detection has the advantage that it avoids ensemble averaging. It was used in a confocal probe volume for the DNA tweezer molecular machine.^126^ For the DNA catenane molecular motor,^98^ TIRF-based single-molecule FRET was used to detect the relative motion of two rings, rotor, and stator, in the motor construct, gaining information on the timing and yield of the motor rotational cycles (Figure 3). To facilitate single-molecule experiments, STED-FRET has been proposed.^129^

### 2.3 Atomic Force Microscopy

AFM is a scanning-probe technique that can image the topology of a surface, on which a specimen is adsorbed, using a tip that ideally is atomically defined. The tip is mounted on a cantilever, the motion of which due to atomic interactions is detected and amplified by a laser beam that is reflected from the cantilever and detected by a quadrant photodetector.^130^ AFM allows one to visualize molecular objects with up to ångström resolution, suitable for nanosized molecular motors. In studies of molecular machines, a tapping mode is typically used, where the cantilever oscillates at a resonance frequency at a constant amplitude set point. The tapping mode is generally more delicate than the contact mode.^130^

The limitation of conventional AFM is generally slow scanning with image acquisition times of minutes and the need for a surface. For many molecular motors, AFM in a liquid is of particular appeal because drying the samples may alter the conformation of the motors and distort motor-substrate complexes. Liquid-phase AFM, in principle, offers a higher resolution than air-phase AFM, as the AFM tip is then not pulled by capillary forces that would act in thin films of water on samples when they are in air (the films are the humidity of the ambient atmosphere).^130,131^ Furthermore, undesirable electrostatic interactions between the tip and sample can be minimized in a liquid by adjusting the ionic strength of the buffer.^131^

**Figure 4:**
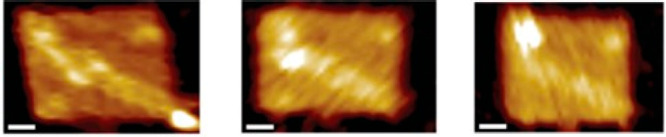
AFM images of a molecular spider (bright dot) moving along a DNA origami, scale bars −20 nm. Reprinted by permission from Springer Nature from ref (67). Copyright 2010.

AFM is especially applicable for motors that move along a track that, by itself, also is detectable by AFM. For example, the motion of single molecular spiders has been imaged (Figure 4) on a DNA origami track^67^ with a nanometer spatial resolution using the tapping mode. High resolution with the tapping mode AFM in a liquid was achieved for the DNA catenane motor,^98^ also walking along a DNA origami track. In another work, using AFM in a liquid, a cargo moved by a DNA robot was imaged over a distance of less than 50 nm.^132^ However, no high temporal resolution was achieved in these works, but AFM images were taken before and after the motion event or with a minutes-scale resolution during the motion.

Of high interest for visualizing motors in real-time is the development of high speed (HS) AFM.^49,50,133,134^ Currently HS-AFM reaches a temporal resolution of 20–100 ms, with 1–3 nm typical lateral resolution, as reviewed in refs (51 and 135). The major factors enabling the high temporal resolution are a small cantilever that operates at high resonant frequencies, a fast scanner, and fast amplitude detector. HS-AFM has already found applications in experiments on artificial molecular motors. For example, HS-AFM was employed to reveal steps of 7.4 ± 1.0 nm of a DNA walker on DNA origami,^71^ using the HS-AFM setup that was described in refs (48 and 49) in real time in a buffer at a frame rate of 0.1 s^–1^. HS-AFM has also been used in a range of studies on natural molecular motors.^136^ The HS-AFM method is still being developed, with the aim to achieve a high spatiotemporal resolution on larger scan sizes^137^ and to measure force.^138,139^ A further increase in speed is achieved by reducing the dimensionality: scanning along a single axis, as fast as ∼1 ms per line, was used to observe an annexin-V trimer rotation with ångström resolution.^140^

Another methodological advancement that is potentially relevant for sensitive motor systems is noncontact AFM (NC-AFM), which allows for completely nondestructive imaging. NC-AFM is based on a cantilever frequency (or amplitude) modulation upon a short-range attractive atomic interaction between the AFM tip and sample.^141^ The tip–sample interactions cause a frequency shift in the cantilever resonance vibration, which is then detected for a reconstruction of the sample topography. By contrast, in tapping mode the cantilever frequency and amplitude are constant. Although cantilevers may exhibit a low *Q* factor in a liquid,^142^ both frequency-modulation and amplitude-modulation AFM have been successfully performed in a liquid. For a frequency-modulation regime in a liquid, a sub-nanometer resolution was achieved for a surface of polydiacetylene^142^ and lipid ion network^143^ on customized setups. In ref (144), the images of lipid bilayers were compared when taken in amplitude- and frequency-modulation regimes, and it was argued that an amplitude modulation is preferable for heterogeneous samples.

### 2.4 Scanning Tunnelling Microscopy

STM has been used for imaging the smallest single-molecule motors,^145^ such as rotary motors. A conductive surface on which the molecular sample is observed, such as Cu(111) or Au(111), serves as an electrode, whereas a nanometer distance between the surface and the scanning tip allows for a tunnelling of electrons under an applied bias voltage.^146,147^ When the surface is not highly smooth, a constant current mode is typically used, which implies that the tunnelling current is kept constant through a variation of the height between the tip and sample.

**Figure 5:**
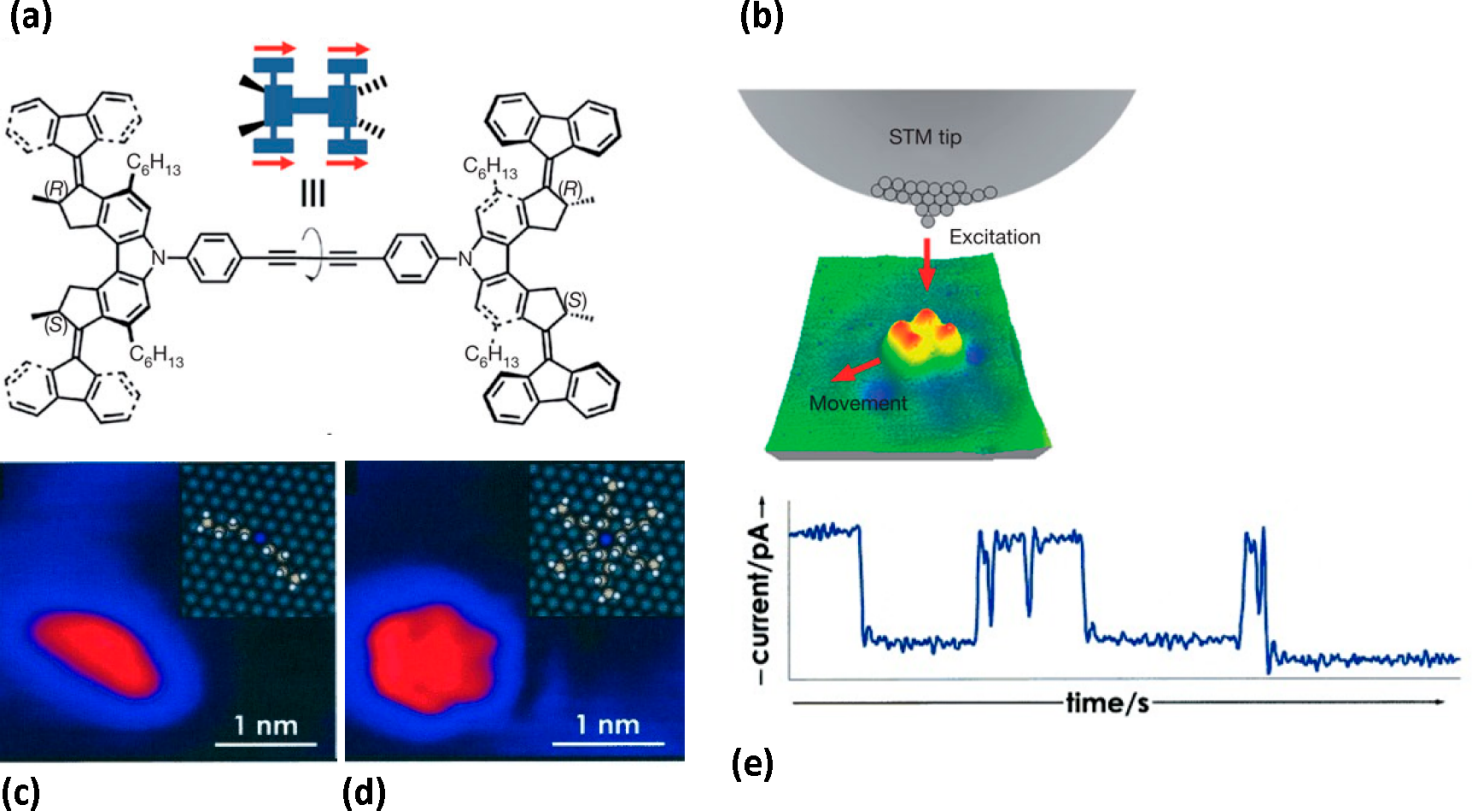
(a, b) Motion of the “four-wheeled” *meso*-(*R*,*S*-*R*,*S*) isomer upon excitation with electrons. Reprinted by permission from Springer Nature from ref (148). Copyright 2011. (a) Structure of the motor and principle of the motion. (*R*) and (*S*) are absolute configurations at stereocenters; the direction of motion of the molecule is shown with red arrows. (b) Schematic of the movement upon excitation from the STM tip. (c–e) STM study on dibutyl sulfide motors. Reproduced from ref (55) with permission from John Wiley and Sons. Copyright 2009. STM images of the motors in a static (c) and spinning (d) state. These states are depicted in insets. (e) Time-dependent tunnelling current, changes in which show the molecular rotation.

STM has also been employed to induce molecular rotation using electrons from the STM tip. The linear motion of a “four-wheeled” motor (Figure 5a) was initiated (with the STM tip being positioned above the motor during excitation, see Figure 5b) and characterized by Feringa and co-workers.^148^ Besides the Feringa motor, STM has been used to drive other molecular “nanocars” that were typically powered either by an electric field gradient created by the STM tip, which attracts/repulses dipoles in a nanocar, or by inelastic electron tunnelling.^72,73^

An excitation with STM electrons was done also for thioether molecular rotors: dibutyl sulfide^55^ (Figure 5c,d), butyl methyl sulfide,^54^ and others.^56^ In these studies, a slow scanning speed (e.g., 1 min per image in ref (54), slower than the molecular rotation) presented a challenge, as multiple orientations of a rotating molecule were superimposed in the images (Figure 5d). However, it was possible to measure the rate of rotation using STM spectroscopy (time-dependent measurements of tunnelling current) (Figure 5e). Another example of high-resolution STM combined with tunnelling current measurements is the imaging of an ångström-sized acetylene motor,^16^ for which current measurements were employed to verify quantum tunnelling. A number of other STM studies on molecular motors are reviewed in ref (145).

Temperature control in an STM setup allows for varying the rate of a nanoscale molecular motion, as done for porphyrin-based system in ref (148). In studies,^54,55^ temperatures below 8 K were necessary to observe static nanoscale motors. Another common prerequisite for STM studies is an ultrahigh vacuum condition, used in refs (55, 148, and 149).

To improve the temporal resolution of STM, fast-scanning instruments have been developed, reaching the imaging rate faster than 0.1 s^–1^.^150,151^ An example relevant for molecular motor studies may be the measurement of diffusion coefficient^152^ for a Violet Lander (C_108_H_104_) molecule. In this study, the diffusion of molecules on a surface was initiated at a temperature of 160–200 K by pushing the molecule with the STM tip. The diffusion coefficient was calculated from an STM movie recorded at a 0.1 s^–1^ framerate.

Thus, the resolution of STM allows for precise imaging, with an additional opportunity to power some nanoscale motors using electrons. However, STM requires conductive substrates as well as additional advancements for faster imaging. STM in liquid is possible, and was used in, for example, ref (153) and, more recently, in ref (154) with a high spatial resolution comparable with that of an ultrahigh vacuum STM. However, that approach remains relatively uncommon.

### 2.5 Optical and Magnetic Tweezers

Because many molecular motors work by exploiting diffusion at the nanoscale, and the thermal energy at room temperature is *kT* ≈ 4 pN nm, the relevant range of forces is on the piconewton scale. Optical and magnetic tweezers (OT/MT) enable force measurements in this range with the required high spatiotemporal resolution (Figure 1 and Table 1). The measurements are performed using trapped micro- or nanobeads, to which the objects of interest are attached, and thus the measured force is applied to these beads. In OT, refractive beads are trapped with a focused laser beam, and the use of a laser-based detection system confers a high spatiotemporal resolution.^155,156^ MT employs super-paramagnetic beads placed in a magnetic field and has the advantage of exerting controlled and constant forces.^157^ OT and MT have been widely used for natural motor experiments, but their use is still uncommon for studying artificial motors.

An experimental design relevant for walker-type motors is a so-called “single bead” configuration. In it, a bead is trapped while attached to a motor that is bound to its substrate. The setup enables one to measure the force generated by the motor when it moves. Recently, this was done with MT for a synthetic DNA walker^69^ to detect a force of 2–3 pN. With OT, this type of trapping has been recently improved^58^ by using germanium nanospheres that allowed for microsecond-scale temporal and nanometer-scale spatial resolution with reduced heating (often an issue of OTs), and it was used to improve the understanding of the stepping process of kinesin.

## 3 Characterization of Molecular Motors in Two-Dimensional Landscapes

Many molecular motors operate without clearly detectable steps, that is, such motors do not dwell for sufficiently long periods of time at a given location. The underlying dynamics of these motors can be particularly difficult to ascertain when their motion is on a two-dimensional (2D) substrate, because there are no steps to be resolved using microscopy, and the large-scale motion can look very similar to thermal diffusion. Insight into the dynamics of these motors can be gained with statistical approaches to a trajectory analysis. In the following, we first introduce a class of such motors and then describe statistical methods for their analysis.

### 3.1 Burnt-Bridges Motors

A burnt-bridges Brownian ratchet (BBR) is a model for a biased molecular motion whereby a particle driven by a thermal motion traverses a lattice of free-energy-rich substrate sites. It cleaves the sites as it moves, thereby releasing free energy and leaving behind a wake of product sites, returning to which is energetically unfavorable. A BBR’s motion is thus biased away from product and toward an energy-rich substrate. While the motion of the BBR is powered purely by random thermal fluctuations (hence “Brownian”), spatial asymmetry resulting from substrate cleavage is essential for the motors to achieve a directed motion.^158^

The performance characteristics of synthetic BBR systems often vary with their polyvalency, that is, with the number of available molecular chemical units (“feet”) capable of binding to, and cleaving, substrate sites.^64,159−161^ Currently, various synthetic BBRs have been realized, both at the nanoscale^1,26,52,67,162−164^ and microscale.^64,65,103^

**Figure 6:**
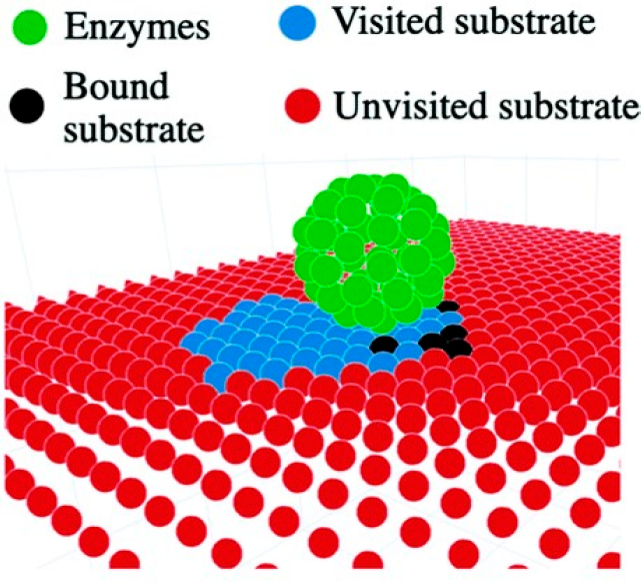
A bead-based burnt-bridges ratchet, enzymatically driven across a 2D substrate. Reproduced from ref (3) with permission. Copyright 2020 Royal Society of Chemistry.

One example of a BBR design that we reference below is the Lawnmower.^165^ It consists of trypsin proteases (protein-cleaving enzymes) tethered to a spherical hub. On a peptide-decorated surface, the Lawnmower is designed to cleave peptides (“blades of grass”), preferentially binding to and cleaving previously unvisited regions. A snapshot of a simulation of a Lawnmower-type motor is shown in Figure 6.

### 3.2 Mean-Squared Displacement Characterization

If the functionality of a molecular motor cannot be verified by observing its individual steps, which is the case for many BBR motors, an anomalous diffusion exponent may serve as an alternative marker. It can be calculated using the mean-squared displacement (MSD), and here we overview both these measures. We outline the applications of MSD for an analysis of trajectories measured in two dimensions (and easily generalizable to three). MSD is a powerful tool used to characterize the dynamical properties of a synthetic system, as it can reveal the extent of superdiffusivity, that is, whether the motor is active and achieves motion beyond simple diffusion. Furthermore, tracking molecular motor motion yields an ensemble of trajectories that for BBR-like designs, in particular, are likely to be heterogeneous by duration, covered distance, etc., and an MSD analysis allows for ensemble studies. An MSD analysis has been used to assess the dynamics of both experimental^1,4,65,77^ and simulated^3,159−161^ systems.

**Figure 7:**
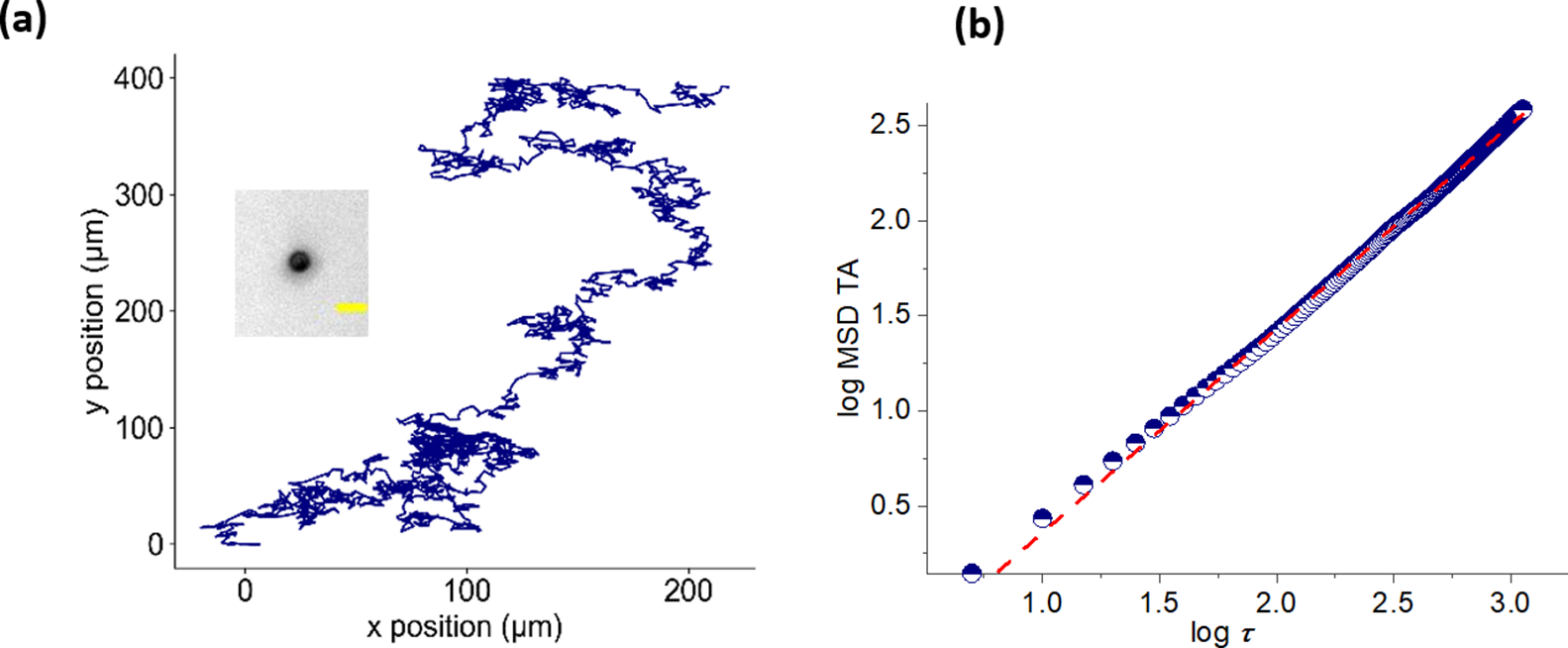
MSD analysis and extraction of anomalous diffusion exponent for a bead-based Lawnmower motor (inset, scale bar 5 μm, see Section 1 of the Supporting Information for details): (a) trajectory of the motor on a cleavable 2D substrate, total duration *T* of the trajectory is 11 265 s, the initial position is (0,0); (b) plot (blue semifilled circles) of log MSD_TA_ (τ) calculated as in (3), as a function of log τ, where the time lag τ is plotted up to 0.1*T* (details of the MSD calculations are in Section 3 of the Supporting Information). The anomalous diffusion exponent α is determined from the linear fit (red dashed line): α = 1.1 (superdiffusive motion).

To compute the MSD for a trajectory in two dimensions (Figure 7a), displacements in *x*(*t*) and *y*(*t*) are used, such as measured from microscopy. In the following paragraphs we define the typical MSD measures used: the single-trajectory time-averaged approach and the ensemble-averaged approach over *N* trajectories. In either case, we define the squared displacement in two dimensions as
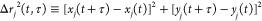
1where *x*_*j*_ and *y*_*j*_ denote the *x* and *y* coordinates of the *j*th trajectory, *t* is time, and τ is the time lag over which the displacement is measured. The minimum time lag is restricted to the frame rate used to image the trajectories in the experiment; for example, if images are collected every 10 s, then the time lag cannot be less than 10 s. The maximum time lag cannot exceed the total duration of the trajectory. The ensemble-averaged (EA) MSD is computed across the entire ensemble of *N* observed particles and is given by
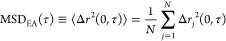
2Equation 2 is useful for characterizing the time-dependent ensemble-averaged diffusion behavior. For example, it can be used to assess if there is a characteristic time to form an asymmetry or if, in the long-time limit, the ensemble of particles loses the ability to move directionally.^3^

For single-particle tracking experiments, the MSD is more commonly computed independently for each particle with a trajectory-averaged (TA) approach. The MSD_TA_ is given by
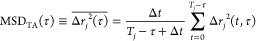
3where Δ*t* is the time increment of trajectory measurements (e.g., interval between frames), and *T*_*j*_ is the total duration of the *j*th trajectory.^166^

With either of these measures, the MSD is related to the dynamics of the system.
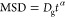
4Here, *D*_g_ is the generalized diffusion coefficient with units of length^2^/time^α^, where *t* is time, and α is the anomalous diffusion exponent. For 0 < α < 1 the system is subdiffusive, meaning that the motion is restricted. For α = 1.0 the system is conventionally diffusive (the ensemble is undergoing a random walk, consistent with Brownian motion), and for 1 < α < 2 the system is superdiffusive (the ensemble has a directional bias, indicating an active motion). For α = 2.0 the system is ballistic (the trajectories are linear). For α > 2 the system is superballistic, whereby the ensemble displays an acceleration over the time scale of interest. A superballistic behavior has been found in artificial molecular systems where the acceleration occurred.^3^ Typically, artificial molecular motors are designed to achieve a superdiffusive motion (1 < α < 2).

Once the MSD is computed from (2) or (3), the anomalous diffusion exponent is estimated from a linear fit to the log of the MSD as a function of log τ (Figure 7b). The type of motion (subdiffusive, diffusive, or superdiffusive) can then be classified over a range of times or time lags. Thus, the MSD allows one to distinguish between superdiffusive functional motors and other moving objects in an experiment.

For systems with 1 < α < 2, in lieu of fitting the MSD to (4), one may fit the MSD to
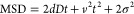
5where *d* is the dimension of the system, *D* is its conventional diffusion coefficient, *v* is velocity, and σ accounts for the experimental position tracking uncertainty. Such an approach has been used for micron-sized artificial molecular machines to quantify their velocity and diffusion coefficient.^103^

Another useful property of the MSD is to assess whether a system is ergodic or nonergodic, where ergodicity is defined as the equivalence between the long-time limit of the ensemble-averaged MSD (eq 2) and the long-time lag limit of the trajectory-averaged MSD (eq 3). Nonequivalence between (2) and (3) in the long-time limit indicates the system is nonergodic.^167^

For noisy systems, errors in particle position detection may result in a misleading MSD analysis, necessitating the development of correction terms.^168,169^ An MSD analysis must also be used with caution for systems with finite processivity (i.e., motors detaching from their substrate after some time of being bound to it) whereby the MSD may report an anomalous behavior despite underlying Brownian dynamics, suggesting the effects of finite processivity need to be deconvolved from the MSD.^166^

## 4 Guided Motion of Artificial Motors

The characterization techniques described so far in this Review have been used to confirm that artificial motor constructs actually work and to obtain quantitative information on the motor performance. Looking ahead, it is of interest to consider nanotechnological applications of artificial motors. One can get an idea of what these may be from the already emerging applications of biological motors, reviewed in refs (170 and 171). These emerging applications include biocomputation,^21,22^ biosensing,^172−174^ diagnostic devices,^175^ and self-assembling structures.^176^ All these devices are based on cytoskeletal microtubules or actin filaments driven by kinesin and myosin. Nanofabricated tracks have been used to guide filament motion, often by a combination of physical confinement by the channel walls and a selective chemical modification of the channel floors.^177,178^

It may thus be beneficial to likewise constrain the motion of artificial motors to one-dimensional tracks. This makes the trajectory analysis easier, may enable future applications, and may lead to an enhanced superdiffusive motion of the motor, as has been shown in simulations of BBRs.^161^ Constraining the motion of artificial motors requires a fabrication of a narrow (quasi-one-dimensional) track. For microscale motors, this has been demonstrated using microcontact printing to fabricate 3 μm wide channels.^65^ On a smaller scale, nanochannels have been fabricated to guide the motion of artificial motors powered by a combination of DNA–protein interactions and salt-induced changes in the DNA conformation.^179^ Alternatively, tracks for engineered biomolecule-based motors have been constructed using DNA origami.^67,98,132^

### 4.1 Quasi-1D Channels for Artificial Burnt-Bridges Motors

**Figure 8:**
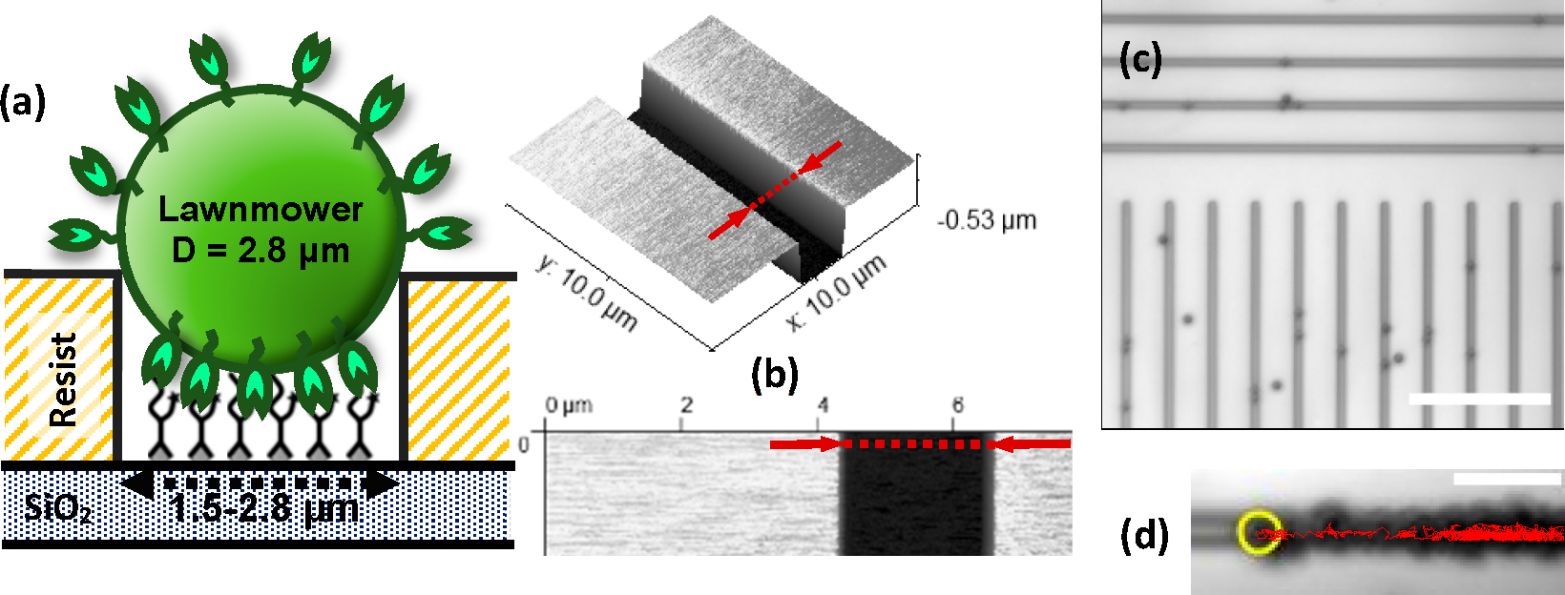
Microchannels for guiding the motion of the Lawnmower BBRs based on microbeads. (a) Cross-sectional schematic (not to scale) of Lawnmower interaction with peptide substrate in a channel; trypsins are depicted as green buds. (b) AFM image of a fragment of a fabricated channel. (c) Lawnmowers (dark spots) on a surface structured with 2.2 μm wide channels. Scale bar is 50 μm. (d) Tracked trajectory (red line) of a single Lawnmower, overlaid on the superposition of the motor images in multiple frames. The yellow circle indicates the initial position; scale bar is 10 μm. For microchannel fabrication details and controls that confirm a selective functionalization, see Section 2 of the Supporting Information.

Here we report on the use of microchannels to guide the motion of polyvalent, microsphere-based BBRs. The motivation is threefold: (i) modeling predicts that channels narrow enough to enforce quasi-1D motion may enhance a superdiffusive motion;^161^ (ii) the one-dimensional motion may facilitate an analysis of diffusive properties; (iii) guided motion along artificial channels would be a key step toward applications of artificial motors. Lawnmowers (polyvalent BBRs; Figure 8a) were fabricated based on ref (180), with 2.8 μm Dynabeads M-270 Amine (Thermo Scientific) serving as motor hubs; for the details of the design and protocol, see Section 1 of the Supporting Information. To constrain the Lawnmower motion, the channel floor (but not the channel walls) was selectively functionalized with the peptide lawn (Figure 8; fabrication details and controls that confirm selective functionalization are reported in Section 2 of the Supporting Information). Microchannels of varying width were fabricated by electron-beam lithography, with a depth chosen such that the bead bottom can interact with peptides on the channel floor. The peptide lawn was supported by a polymer brush (F127), which effectively blocks a nonspecific binding to the underlying surface.^181^

**Figure 9:**
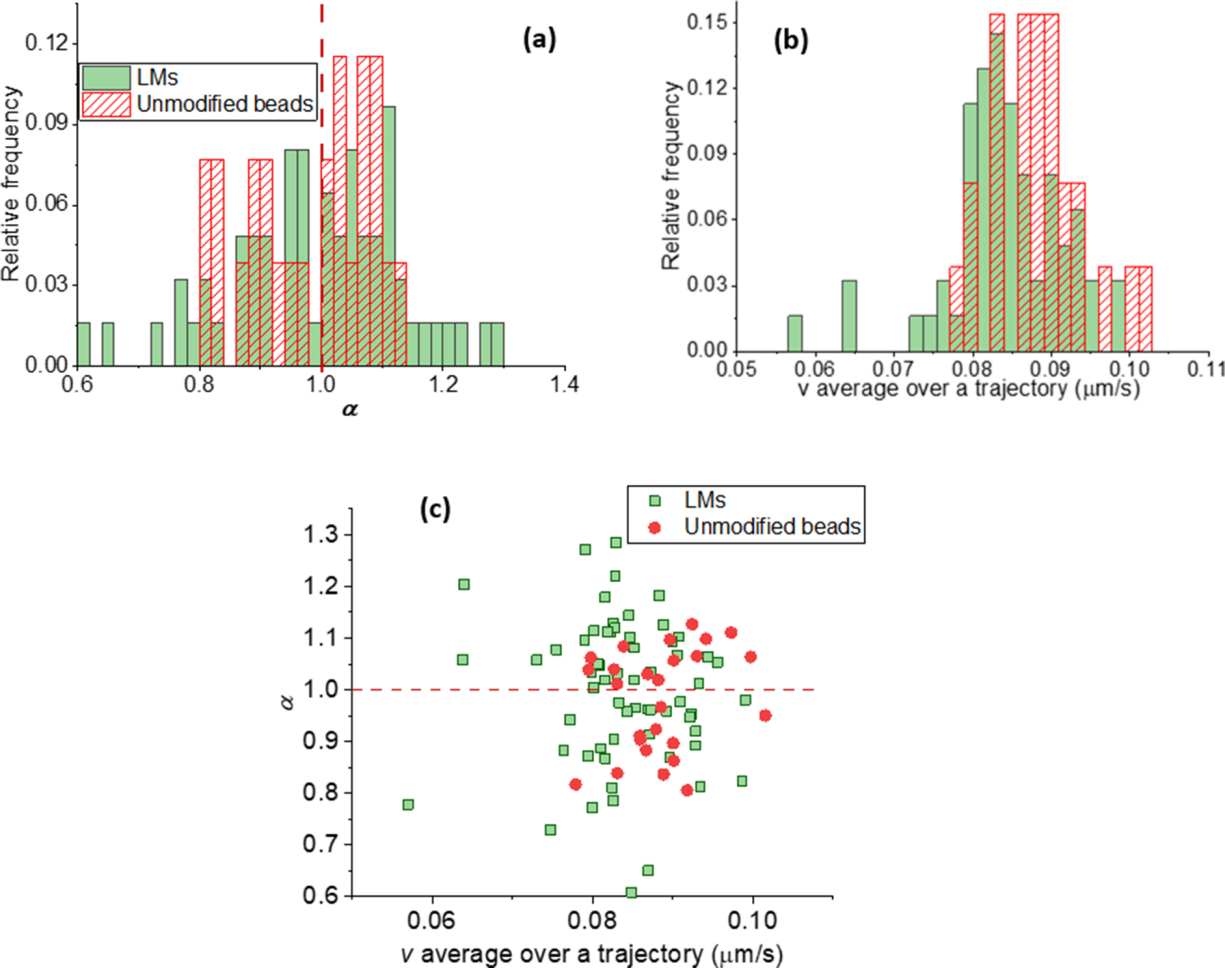
Anomalous diffusion exponents (a), average speeds over 10 s intervals (b), and correlation of these (c) for the Lawnmowers (LMs) (Figure 8) in 2.2 μm wide channels with the peptide substrate cleaved by proteases on the motors and for unmodified beads (i.e., not motors) in the channels without the peptide cover.(b, c) Stalling for a fraction of the Lawnmowers.

In Figure 9a, we show anomalous diffusion exponents α for the motors in 2.2 μm wide, 0.5 μm deep channels and for unmodified beads (i.e., not motors) in channels with no peptides but only polymer-brush (F127) lawns. The mass density of the used beads allows them to sink in the buffer solution and get confined to channels by gravity. As a result, one would expect 1D diffusion. The exponents were calculated from MSD_TA_ (eqs 1 and 3), and the calculation details are described in Section 3 of the Supporting Information. Not plotted here are low-motile objects (both motors and unmodified beads), which are so classified by very low α < 0.6 and/or speed *v* < 0.05 μm/s (average over a trajectory).

For motile unmodified beads we see (Figure 9a) a distribution of α centered around α = 1, which demonstrates a normal diffusion. This result also shows that the channels can be used to guide the motion, because gravity, which facilitates confinement of the motors within channels, does not hinder the diffusive motion of the unmodified beads.

For the Lawnmowers on the peptide substrate, we observe more heterogeneous dynamics. A handful of them on peptide lawns have α > 1 indicating a superdiffusive motion (see Section 3.2), but there is no strong preference for these trajectory-averaged dynamics when compared with unmodified beads in the channels. A fraction of the Lawnmowers on peptide lawns shows α < 0.8, while no unmodified beads demonstrate that extent of subdiffusive motion. That indicates a hindered diffusion of the motors. Studies of Lawnmower dynamics on 2D substrates^182^ indicate that this design spends a significant fraction of time in a locally entrapped state, even though superdiffusive bursts do occur, as expected from the Lawnmower design.

Surprisingly, the average speed *v* over a trajectory (Figure 9b) is not greater for the motors on the peptide substrate than for unmodified beads in channels with polymer-brush lawns lacking peptides. The average *v* for Lawnmowers in these 1D channels is similar to that observed on 2D peptide lawns^66^ suggesting that similar dynamics are occurring in this constrained geometry.

In 2.8 μm wide channels, we observed similar results (Figure S 2) as in 2.2 μm wide ones. This allows us to conclude that the Lawnmowers in the channels may exhibit a superdiffusive motion, although the motion is hindered for a fraction of the motors, likely because of stalling on the substrate.

In the future, microfabricated or similar patterned structures can be used for guiding molecular motors for a targeted delivery of cargos such as liposome vesicles^183^ and microstructures.^124^ There is a development of nanodevices with nanofabricated structures used for biocomputation when explored by the guided movement of cytoskeletal filaments propelled by molecular motors.^21,22^ Directional movement of bead-based motors could be an alternative to the filaments in performing the computation. Channels can also serve as “testing grounds” for artificial motors, where effects of confinement are studied, for example, for bead-based motors of varying size, for which narrower channels can facilitate the directional bias^161^ but also may decrease the binding footprint. Moreover, a fabrication can be further advanced to three-dimensional structures using lithography techniques such as two-photon polymerization (2PP)^184^ that employs gravity to guide the motors. The 2PP technique employs highly focused femtosecond laser pulses on photocurable and biocompatible resins such as ORMOCER^184,185^ to create 3D structures that confine and guide the movement of motor beads. Another possible technique would be directed 1D motion through tailor-made hollow nanowires, which are demonstrated^186^ to be faster than diffusion for motor-driven transport. When fabricated on reflective surfaces, 3D structures alter the fluorescence interference contrast (FLIC), which in the field of molecular motors has been used for tracking microtubules on kinesin-coated three-dimensional surfaces with nanometer precision;^187^ this can potentially be used also for the tracking of labeled artificial motors.

### 4.2 Nanowires for Optical Observation of Diffusive Molecular Motors

Nanowires (NWs) are cylindrical structures with a diameter on the order of 100 nm and a high length-to-diameter ratio. The development of semiconductor NWs in the context of biosensing^188−192^ and molecular-motor-based assays^193^ is motivated by their ability to act as waveguides: due to the high refractive index of their material, NWs can behave as nanoscaled optical fibers in the visible wavelength range.^194^ Fluorescence generated close to their surface can excite the supported waveguide modes, resulting in the emitted photons being guided to their tip.^195−197^ Thus, NWs act as optical integrators and may also enhance the excitation and emission of nearby dyes; this results in an enhanced fluorescence.^192,193,198,199^ Gallium phosphide (GaP) NWs are particularly advantageous because the high refractive index of GaP^200^ enables lightguiding already at small (100 nm) diameters, and they do not absorb light in the visible range greater than 460 nm due to their bandgap.^201^ Additionally, these NWs have been shown to be biocompatible.^202,203^

Fluorescence enhancement and guiding have been proven useful for tracking actin filaments on myosin-coated GaP NWs,^193^ where the fluorescence of labeled filaments was enhanced by the NWs and conveniently observed at the NW tip. Furthermore, this signal was observed to change when a motor was moving along the NW. This approach has also been proven effective to measure the diffusion of smaller molecules, specifically cholera toxin subunit B in a lipid bilayer deposited on the NWs.^191^ The NW tips would “blink” whenever a protein diffuses onto the surface.

These studies indicate that NWs might be useful for characterizing a mechanochemical coupling of nanoscale artificial motors. As an example, we herein outline recent progress made with a quantum-dot-based Lawnmower motor, the design of which is similar to that we developed (Figure 8a) for guiding in channels (Section 4.1) but employing a quantum dot instead of a bead; this construct was conceptualized in ref (165).

**Figure 10:**
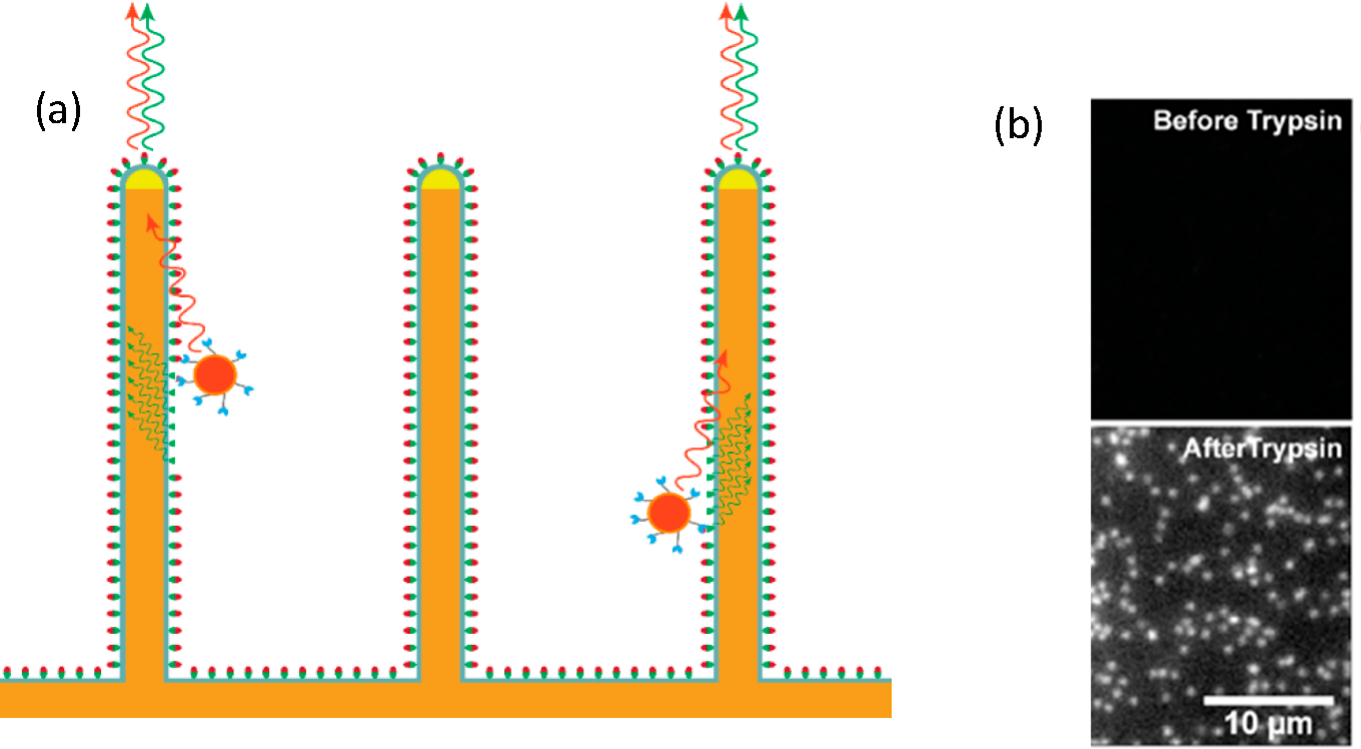
(a) Schematic of a proposed experiment with quantum-dot (depicted in red) Lawnmowers. Quantum-dot Lawnmower motors are moving along the surface of NWs, activating fluorophores as each NW-bound substrate site is cleaved. The signal is then guided to the tips of the NWs and is detected as well as the signal from the quantum dots. (b) Result of trypsin-induced substrate cleavage on the GaP NWs (see Section 5 of the Supporting Information for the experimental details).

Since a BBR such as the quantum-dot Lawnmower requires a cleavable substrate, it is convenient to make the substrate fluorogenic, so that it becomes fluorescent upon cleaving (Figure 10; the fluorogenic substrate for Lawnmowers is described in Section 2 of the Supporting Information and in ref (181)). If the NWs surface is functionalized with the substrate and the signal of the substrate and quantum dot are in different wavelength ranges, it will be possible to confirm both the motor presence and activity by monitoring the light emitted from the NW tips. The density of NWs would enable hundreds of observations in parallel. The motor’s behavior could then be characterized by correlating the time spent on an NW with the intensity of the generated cleavage signal.

To create on NWs a landscape suitable for the motor, we have been developing surface chemistry protocols to attach the fluorogenic peptide substrate^165^ to the surface of silicon-coated waveguiding GaP NWs combining different click-chemistry protocols.^180,181,204^ The details of the functionalization are described in Section 5 of the Supporting Information. While the peptide has been attached reliably on the NWs, the surface chemistry remains to be optimized in order to ensure the motor only interacts with the peptide.

## 5 Outlook and Conclusions

Successful development of artificial molecular motors requires techniques that can capture their motion. Once a motor design is proven functional, it may be optimized and eventually employed for applications. Optical microscopy, AFM, and FRET are arguably the most versatile methods for detecting the lateral motion of individual motors. These three groups of techniques have already been applied to various sizes of motors and in a liquid phase, which is often necessary for the motors to operate. For nanoscale motors, in particular, rotary motors, STM has been commonly employed.

Conventional optical microscopy, either label-free or based on fluorescence, can reach a millisecond-scale temporal resolution. Whereas its spatial resolution is diffraction-limited, a fluorophore localization allows a detection of single motors with nanometer accuracy. Several emerging approaches are currently pushing the boundaries of optical microscopy; these include computational solutions for resolution improvement and denoising, high-speed cameras, fluorophores with increased photostability, and the development of label-free modalities. As a result of these developments, optical setups will most probably remain the most accessible tool to visualize the dynamics of molecular motors.

The diffraction limit is circumvented by fluorescence-based super-resolution techniques that make use of switchable fluorophores (STED, STORM, MINFLUX, and the like), DNA hybridization (DNA-PAINT), or advanced optics (SIM). Currently, these methods have had limited application in artificial motor studies, but they are very probable to be widely employed for nanoscale motors in the future. Although the temporal resolution of, for example, STORM and DNA-PAINT presents a limitation for a real-time tracking of moving motors, state-of-art MINFLUX or video-rate STED would allow tracking with a high temporal resolution. All super-resolution techniques employ computational image reconstruction, and further development in this area, likely due to a wider implementation of GPU calculations and deep learning, may improve the resolution.

AFM offers sub-nanometer resolution, but it comes at the price of more challenging sample preparation, because a surface is needed, and, for many motors, imaging must be carried out in a liquid. Furthermore, conventional AFM has a low temporal resolution, and, from a practical side, the advanced setups are generally less accessible than optical microscopes. Further development of high-speed AFM in liquid and noncontact AFM holds great promise for future motor studies. STM also requires a surface that additionally needs to be conductive, while the key advantages of this method are an outstanding spatial resolution and the possibility to manipulate the motors using an STM tip (electron emission from the tip, movement). Like AFM, STM requires an advanced setup for an image acquisition faster than on the minutes scale.

For motors moving not along a track but on a 2D surface, the trajectories obtained by microscopy need to be analyzed in detail to extract information about the motor performance. We provide an outlook, illustrated by our own results for Lawnmowers, protease molecular motors, on how MSD for an ensemble and a single trajectory can be used to estimate linearity and processivity of motion.

Measurements on individual molecular motors present a challenge beyond just seeing and characterizing the motion. Motor studies often involve FRET because labeling the motors and/or their substrate with a FRET pair of fluorophores allows for a detection of molecular interactions through distance measurements. Single-molecule FRET, already being used in motors research, has the potential to replace ensemble-average FRET measurements for motors.

As the performance of artificial motors increases, force detection methods will become increasingly important. Detailed studies of artificial motors with known design principles may shed light on the fundamental mechanisms of force generation in natural motors, such as the interplay of a “power stroke” and Brownian diffusion.^34,205^ Furthermore, one must know the generated forces to use motors for applications, for example, for cargo transportation. One strategy for force measurements relies on force markers in the sample. Tension-gauge tethers with a known rupture strength were used to that end for a bead motor in ref (64). An inclined surface was used in ref (206) to estimate the action of rotaxane motors, the photoinduced switching of which was moving a macroscopic droplet against viscous forces and gravity. The high resolution of force measurements that is possible with optical and magnetic tweezers (Table 1) makes these methods likely to be applied more widely to synthetic motors in future.

In addition to the experimental challenges of high-resolution tracking, data extraction, and miniaturization of measurements, the directionality of motion along a defined trajectory is yet to be achieved for most artificial motors. For molecular walkers, the directionality is often due to the guidance of the motor along a polymeric or similar molecular track. An alternative, widely used with natural motors but largely unexplored for artificial motors, is to guide the motors along a micro- or nanostructure. Compared to molecular tracks, fabricated tracks have the advantage of a controlled positioning of a track in a sample and of motors on the track as well as a larger variety of functionalization options. Nanowires (or similar high aspect ratio nanostructures) and microchannels are two candidates that can be used for this purpose, possibly in the way that we outline in this Review.

## Supporting Information Available

Construction of lawnmowers, fabrication and functionalization of microchannels, tracking of microscale lawnmowers. Functionalization of nanowires (PDF)

## List of Abbreviations

AFM: atomic force microscopy
ALEX (FRET): alternating laser excitation (FRET)
BBR: burnt-bridges Brownian ratchet
DNA-PAINT: DNA points accumulation for imaging in nanoscale topography
FIONA: fluorescence imaging with one-nanometer accuracy
FPALM: fluorescence photoactivation localization microscopy
FRET: Förster resonance energy transfer
OT: optical tweezers
MT: magnetic tweezers
PALM: photoactivated localization microscopy
PIE: pulsed interleaved excitation
SIM: structured illumination microscopy
STED: stimulated emission depletion (microscopy)
STM: scanning tunnelling microscopy
STORM: stochastic optical reconstruction microscopy
TIRF microscopy: total internal reflection fluorescence microscopy
