# Supplementary material for: Through the Eyes of Creators: Observing Artificial Molecular Motors
Source: ACS Nanosci Au. 2022 Jan 13;2(3):140–59. doi: 10.1021/acsnanoscienceau.1c00041 (PMC9204826; doi:10.1021/acsnanoscienceau.1c00041)
Supplement: Supplementary file 1 — ng1c00041_si_001.pdf [file ng1c00041_si_001.pdf]

Supplementary Information

Through the eyes of creators: observing artificial molecular motors

Ivan Unksov [1], Chapin S. Korosec [2], Pradheebha Surendiran [1], Damiano Verardo [1,3], Roman Lyttleton [1], Nancy R. Forde [2], Heiner Linke [1]*

[1] Solid State Physics and NanoLund, Lund University, SE-221 00 Lund, Sweden

[2] Department of Physics, Simon Fraser University, Burnaby, British Columbia, Canada V5A 1S6

[3] Aligned Bio AB, Medicon Village, Scheeletorget 1, 223 63 Lund, Sweden

* heiner.linke@ftf.lth.se

Section 1. Construction of microsphere-based Lawnmowers

The procedures to assemble Lawnmowers are based on those described in [1,2]. All the solutions were prepared using MilliQ water.

For functionalization of Dynabeads™ M-270 Amine (Thermo Scientific, catalogue number 14307D), we used trypsin thiolated with TCEP according to the following procedure. Freshly thawed 50 µL of trypsin (from bovine pancreas, Sigma-Aldrich) 10 mg/mL in 1 mM HCl was dissolved with 450 µL of buffer (sodium phosphate 0.1 M pH 7.6 or MOPS 0.02 M pH 7.4, Sigma-Aldrich). 2-2.5 mL of Pierce™ Immobilized TCEP Disulfide Reducing Gel (Thermo Scientific, catalogue number 77712), warmed to room temperature, were centrifuged at > 1000 G for 1 minute, and the supernatant was exchanged to the buffer. This procedure was repeated three times, after which TCEP gel sediment was collected. The trypsin solution was added to the TCEP gel sediment, and the mixture was incubated for 30-60 minutes on a rotary mixer. The incubation was followed by centrifugation (> 1000 G, 3 minutes) to sediment the TCEP gel, and thiolated trypsin was recovered from the supernatant.

Thiolation was verified by an absorbance measurement in a sample of Ellman's reagent (stock concentration 10 mg/mL in sodium phosphate buffer 0.1 M pH 7.4), trypsin and the buffer used above, in a volume ratio 1:10:11 respectively.

Functionalization protocol involved multiple sedimentations of magnetic beads, done with a magnet. 50 µL of Dynabeads™ M-270 Amine (stock concentration $2x10^9$ beads/mL) were washed by sedimenting the beads at least two times in 1.5 mL of a buffer (for functionalization, we used 0.02 M MOPS pH 7.4), and changing the supernatant to the fresh buffer. 2 mg Sulfo-SMCC (Thermo Scientific, catalogue number A39268), freshly dissolved in 300-500 µL of the buffer, were added to the beads sediment. At least 2 hours long incubation was carried out on a rotary mixer to avoid sedimentation. The beads were then washed from excessive Sulfo-SMCC by sedimenting at least 4 times in the refreshed buffer. After that, the beads were incubated for at least 4 hours on the mixer with 500 µL of 1 mg/mL thiolated trypsin, then washed by sedimenting in 2 mL of the refreshed buffer at least 5 times, and finally resuspended in the buffer for imaging.

Section 2. Fabrication and functionalization of microchannels to guide artificial molecular motors

Binding and guidance of the Lawnmower motors in peptide-coated channels required the peptide substrate to be deposited selectively on channel floors. As described in [3], NHS(*N*-Hydroxysuccinimide)-modified peptides can be attached to a brush of NHS-labelled Pluronic F127 polymers which are in turn deposited on a hydrophobic surface. We used the protocol and peptides as in [3] to functionalize the channels, however we achieved hydrophobicity in a different way than with the method described there for planar glass surfaces. Below we outline the fabrication of channels, and how the selective hydrophobicity of the channel floors was achieved. Additionally, for a reference sample where unmodified beads (i.e., not Lawnmowers) were used (Figure 8), we made channels functionalized with F127 without the NHS group, and thus with no peptide substrate.

A Si substrate with a thermally oxidized SiO_2 layer of 100 nm thickness was prepared for fabricating the microchannels. The substrates were cleaned in acetone and isopropanol in the ultrasonic bath for 5 min each at room temperature. They were then dried under nitrogen flow and were subjected to plasma cleaning to remove the organic debris. The substrate was spin-coated at 2500 rpm with CSAR 62 (Allresist GmbH, Strausberg, Germany) to a thickness of 500 nm and baked on a hotplate at 180°C for 120 s. The microchannels were patterned by electron beam lithography (Voyager, Raith GmbH, Dortmund, Germany) with a dose of 250 $\mu C/cm^2$. The fabricated substrates were developed using the developer amyl acetate by immersing the chips for 90 s and rinsed in IPA for 30 s, followed by drying under nitrogen flow.

Floors of the channels were made hydrophobic, while the walls were made hydrophilic and thereby not supporting the Pluronic F127 attachment. For that, the developed substrates with channels were first oxygen plasma ashed (Plasma Preen II-862, Plasmatic Systems, Inc., North Brunswick, NJ) at 5 mbar for 30 s which would make the CSAR 62 resist hydrophilic thereby making the surface chemistry different between the exposed channel floors and the resist walls[4,5]. The substrates were then silanized with trimethylchlorosilane (TMCS), which modifies the hydrophobicity of the surfaces, in a controlled chamber at 200 mbar to realize desired contact angle. The contact angles were measured on the controls of planar (i.e., without channels) SiO_2 treated with TMCS. These controls had no resist on them. Attachment of NHS-labelled Pluronic F127 polymers on the surfaces with different contact angles were tested. The desired attachment was found on the TMCS treated SiO_2 surface with a contact angle of 90±2 degrees.

Furthermore, we used NHS-modified fluorogenic peptides, and verified selective functionalization of a test channel (wider than the channels we used in motor experiments) using fluorescence (Figure S 1).

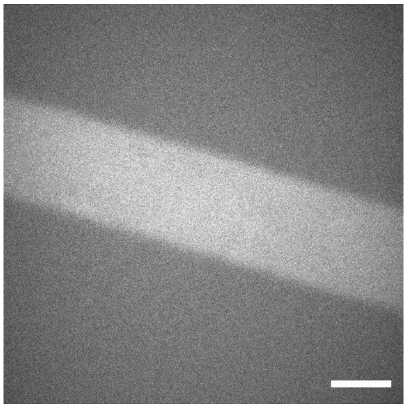

Figure S 1. Fluorescence of peptides at the floor of a test channel. Scale bar is 50 µm.

Section 3. Tracking microscale Lawnmowers and trajectory analysis

We tracked the Lawnmowers within the channels using ImageJ plugin MTrack2 (http://imagej.net/MTrack2). We verified by observation that the motors which went out of the channels or collided with other motors were no longer tracked after such events. MSDs$_{TA}$ were calculated so that the time lag τ did not exceed $0.1T_j$ where T_j is the total duration of the jth trajectory. Anomalous diffusion exponents shown in were calculated as the slope of $\log \mathrm{MSD}$ ($\log \tau$):

$$\alpha = \frac{d(\log \mathrm{MSD})}{d(\log \tau)}.$$

Speeds of unmodified beads and Lawnmower motors were calculated as:

$$v_j(t,\tau) \equiv \sqrt{[x_j(t+\tau) - x_j(t)]^2 + [y_j(t+\tau) - y_j(t)]^2},$$

where j is the number of the trajectory, τ is time lag. For calculation of speeds we assumed $\tau =$ 10 s which is sufficient to exclude from our analysis small fluctuations of the bead position. Next, the speeds were averaged over each trajectory to be presented in Figure 8b and Figure S 2b.

Section 4. Behaviour of Lawnmowers in channels with 2.8 μm width, 0.5 μm depth.

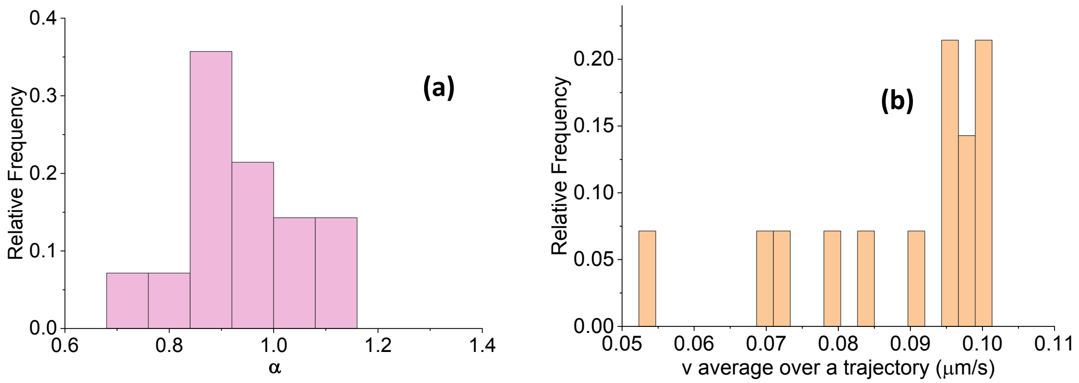

Figure S 2. Anomalous diffusion exponents (a) and average speeds (b) for Lawnmowers in 2.8 μm wide channels with the peptide substrate.

Section 5. Functionalization of nanowires

Silicon-coated nanowires were functionalized with azido-PEG_4-peptide via copper(I)-catalyzed azide-alkyne cycloaddition (CuAAC). Nanowires were first functionalized in vapour phase with an alkyno-silane (O-(propargyl)-N-(triethoxysilyl propyl)carbamate, OTPC for short), using a similar, nonreactive silane ((triethoxysilylpropyl)carbamate, TPC for short) as control, then they were incubated in water phase with the azido-PEG_4-peptide. Ascorbic acid and copper(II)sulfate were used to generate the copper(I) ions necessary to catalyze the reaction.

Figure S 3 displays the results of a trypsin proteolysis assay. Before trypsin was injected into the flow cell, a dark background was observed (Figure S 3a, top). After 100 s following the trypsin injection, we observed an array of light-active nanowire tips (Figure S 3a, bottom). Figure S 3b displays the average time-dependent signal of nanowire tips: the intensity can be seen to increase from background to a maximal value before a time-dependent decrease. The time-dependent decrease is typical for bleaching fluorophores [6].

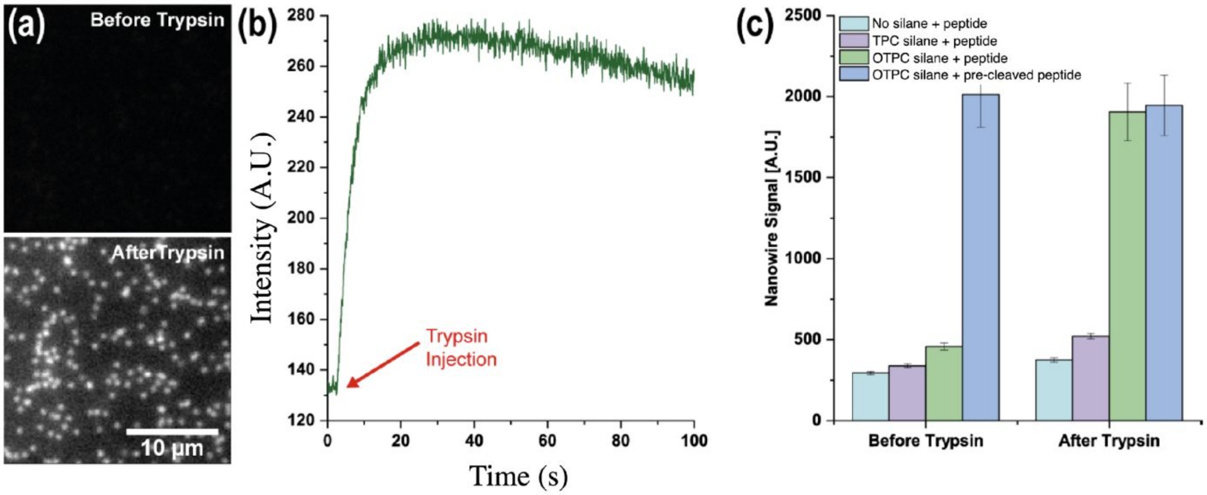

Figure S 3. Cleavage of specifically bound peptide on functionalized nanowires. (a) Fluorescence micrograph of a nanowire sample before and after being exposed to trypsin. (b) Signal from a single nanowire over time, after exposure to trypsin (0.1 mg/mL in PBS pH 8). (c) Average signal from nanowires before and after exposure to trypsin. The two control samples, one without any silane, the other functionalized with the unreactive silane TPC, were incubated with the peptide, but showed no significant increase after trypsin exposure. The sample functionalized with the alkyne-silane OTPC and peptides shows a large signal increase after trypsin activation, close to the signal measured on a sample functionalized with OTPC and pre-cleaved peptide.

Figure S 3c summarizes the proteolysis results across the click-chemistry surface treatments and controls. The OTPC-peptide nanowire sample incubated with trypsin was found to climb to the same signal as the pre-cleaved (fluorescent) peptide control. Nonspecific binding of the peptide was minimal, as shown by the TPC silane and no-silane controls where minimal signal increase was observed upon trypsin injection. These controls suggest that we successfully bound our peptide to the nanowire surface with minimal nonspecific binding, and furthermore, the bound peptide was proteolytically accessible to trypsin.

Although the attachment and cleavage of peptides on NWs worked seemingly well (Figure S 3), we found that amine-functionalized quantum dots are abruptly and permanently adsorbed to the NW surfaces (Figure S 4, yellow circles), when we flowed in 50 µl of 5 mM amine quantum dots in PBS pH 8 buffer.

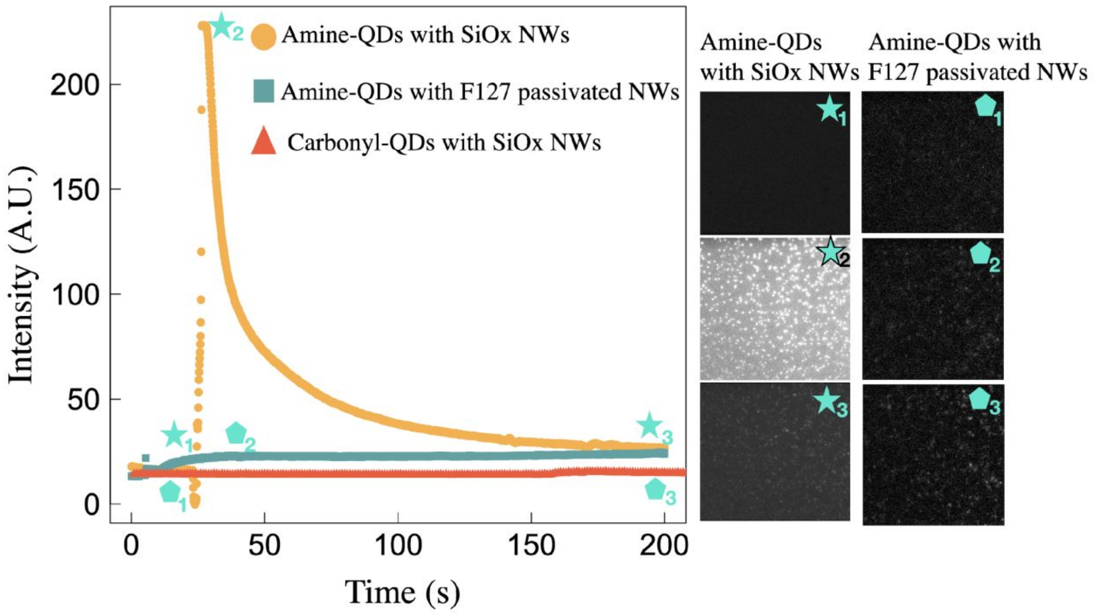

Figure S 4. NWs passivated with F127 block non-specific binding of amino quantum dots. Left: Amino quantum dots (5 mM) bind to NWs treated with silicon oxide deposition and ozone plasma immediately following their injection into the microfluidic chamber (yellow circles). 5 mM of carbonyl quantum dots yields no adsorption (red triangles). NWs passivated with the F127 polymer brush and subject to 5 mM amino quantum dots experienced a slow increase in binding (blue squares). Right: various snapshots of the NW arrays to visualize intensity of quantum dot binding. Time of snapshots is indicated by the corresponding symbol on Intensity plot.

We therefore implemented a tri-block copolymer chemistry using Pluronic F127 previously shown to block non-specific particle interactions[6]. We then flowed in 50 µl of 5 mM amine quantum dots in PBS pH 8 buffer into a sample of F127-passivated NWs and found that adsorption was dramatically slower (Figure S 4, blue squares) for NWs coated with F127 than for bare SiOx NWs. To test if electrostatic interactions were responsible for the binding of (positively charged) amine quantum dots to the NWs, we used an equal amount of (negatively charged) carbonyl quantum dots in a control assay. We found that throughout the 200 s duration of the experiment no carbonyl quantum dots bound to the surface of NWs, as evidenced by a lack of any increase in fluorescent signal at the NW tips (Figure S 4, red triangles).

References

(1) Kovacic, S.; Samii, L.; Curmi, P. M. G. G.; Linke, H.; Zuckermann, M. J.; Forde, N. R. Design and Construction of the Lawnmower, an Artificial Burnt-Bridges Motor. *IEEE Trans. Nanobioscience* **2015**, *14* (3), 305–312. https://doi.org/10.1109/TNB.2015.2393872.

(2) Korosec, C. S.; Forde, N. R. The Lawnmower: An Artificial Protein-Based Burnt-Bridge Molecular Motor. *arXiv: physics.bio-ph/2109.10293* **2021**.

(3) Kirkness, M. W. H.; Korosec, C. S.; Forde, N. R. Modified Pluronic F127 Surface for Bioconjugation and Blocking Nonspecific Adsorption of Microspheres and Biomacromolecules. *Langmuir* **2018**, *34* (45), 13550–13557. https://doi.org/10.1021/acs.langmuir.8b02877.

(4) Bunk, R.; Sundberg, M.; Månsson, A.; Nicholls, I. A.; Omling, P.; Tågerud, S.; Montelius, L. Guiding Motor-Propelled Molecules with Nanoscale Precision through Silanized Bi-Channel Structures. *Nanotechnology* **2005**, *16* (6), 710–717. https://doi.org/10.1088/0957-4484/16/6/014.

(5) Lindberg, F. W.; Norrby, M.; Rahman, M. A.; Salhotra, A.; Takatsuki, H.; Jeppesen, S.; Linke, H.; Mansson, A. Controlled Surface Silanization for Actin-Myosin Based Nanodevices and Biocompatibility of New Polymer Resists. *Langmuir* **2018**, *34* (30), 8777–8784. https://doi.org/10.1021/acs.langmuir.8b01415.

(6) Kirkness, M. W. H.; Forde, N. R. Single-Molecule Assay for Proteolytic Susceptibility: Force-Induced Collagen Destabilization. *Biophys. J.* **2018**, *114* (3), 570–576. https://doi.org/10.1016/j.bpj.2017.12.006.
